# Transcriptomic and proteomic profiling identifies feline fibrosarcoma as clinically amenable model for aggressive sarcoma subtypes

**DOI:** 10.1016/j.neo.2024.101104

**Published:** 2024-12-15

**Authors:** Mikiyo Weber, Daniel Fuchs, Amiskwia Pöschel, Erin Beebe, Zuzana Garajova, Armin Jarosch, Laura Kunz, Witold Wolski, Lennart Opitz, Franco Guscetti, Mirja C. Nolff, Enni Markkanen

**Affiliations:** a Institute of Veterinary Pharmacology and Toxicology, Vetsuisse Faculty, University of Zurich, 8057 Zürich, Switzerland; b Institute of Pathology, Charité-Universitätsmedizin Berlin, 10117 Berlin, Germany; c Functional Genomics Center Zürich, ETH Zürich/University of Zurich, 8057 Zürich, Switzerland; d Institute of Veterinary Pathology, Vetsuisse Faculty, University of Zurich, 8057 Zürich, Switzerland; e Clinic for Small Animal Surgery, Vetsuisse Faculty, University Animal Hospital, University of Zurich, Zurich, Switzerland

**Keywords:** Soft-tissue sarcoma, Comparative oncology, Disease model, Tumor targeting, Laser-capture microdissection, RNAsequencing, LC-MS/MS, Feline injection-site sarcoma

## Abstract

Fibrosarcomas (FSA) are malignant mesenchymal tumors characterized by low chemo- and radiosensitivity. Development of novel treatment strategies for human adult FSA is hindered by the low incidence and the absence of suitable clinical models. Interestingly, aggressive FSA occur more frequently in domestic cats, hence potentially representing a clinically amenable model to assess novel therapies such as targeted imaging or theranostics. However, a lack of molecular characterization of FSA and adjacent normal tissue (NT) in both species hinders identification of tumor-specific targets and undermines the translational potential of feline FSA. Combining laser-capture microdissection, RNAsequencing and liquid chromatography-tandem mass spectrometry, we perform comprehensive profiling of 30 feline FSA and matched skeletal muscle, adipose and connective tissue. Clear inter-tissue differences allow identification of significantly upregulated and tumor-exclusive features that represent potential targets for diagnostic and therapeutic approaches. While feline FSA are characterized by hyperactive EIF2, TP53 and MYC signaling, immune-related and neuronal pathways emerge as modulators of tumor aggressiveness and immunosuppression. A high degree of molecular similarity with canine and adult FSA allows identification of tumor targets that are conserved across species. Significant enrichment in DNA repair pathways in feline FSA correlate with aggressive clinical behavior in human soft-tissue sarcoma. Finally, we leverage the molecular profiles to identify vulnerabilities, including sensitivity to ATR and PARP inhibition as potential treatment for feline FSA. In conclusion, this detailed landscape provides a rich resource to identify target candidates and therapeutic vulnerabilities within and across species and supports feline FSA as relevant models for the human disease.

## Introduction

Fibrosarcomas (FSA) are tumors of mesenchymal origin that affect humans and cats alike [1,2]. In both, FSA are characterized by locally aggressive growth and tissue invasion that result in high rates of tumor recurrence as well as low sensitivity to radio- and chemotherapy [2,3]. In humans, FSA are classified as ‘infantile FSA’ and ‘adult FSA’. Adult FSA are very rare, highly malignant tumors with a poor prognosis [4]. They are diagnosed by exclusion of other soft-tissue sarcoma subtypes [4] and their low incidence is challenging in many aspects, ranging from insufficient understanding of the underlying pathological mechanisms and genetic aspects, absence of diagnostic markers, to a lack of efficient therapies for affected patients. Hence, clinical progress in treating FSA is undermined by the small number of affected patients, a situation that is further exacerbated by the absence of valid disease models.

Interestingly, naturally developing tumors in domestic animals present potentially valuable models to further develop our understanding of tumor biology and treatment. By integrating naturally occurring cancers observed in veterinary patients into the analytic and developmental pipeline for novel therapies, comparative oncology aims to improve cancer management [[5], [6], [7]]. Comparative oncology is particularly promising for FSA: while exceedingly rare in humans with an incidence of around 0.2 cases in 100,000 [8], FSA in domestic cats are frequent, representing between 12-41 % of all feline cutaneous tumors [9,10]. Clinically, feline FSA represents an aggressive, infiltrative tumor type prone to local recurrence and with a metastasic rate between 10 and 24 % [11]. As such, the combination of high prevalence, similar histomorphology and clinical behavior suggests feline FSA as highly interesting as naturally occurring and clinically amenable models for adult FSA. Identification of molecular homology between human and feline FSA could therefore facilitate development and structured clinical assessment of novel therapies that are difficult or impossible to assess in the human setting, while simultaneously unlocking novel treatment options for affected cats.

Feline FSA is currently classified into two possible entities, namely feline injection site sarcoma (FISS) – the more aggressive and frequent form - and non-injection site FSA. FISS is a malignant tumor that originates from the excessive growth of fibroblasts and myofibroblasts in regions of persistent inflammation, especially at injection sites [12,13]. Although the association between FISS and vaccines is strongest, other medical procedures that involve injection, such as the use of long-acting steroids and antibiotics, nonabsorbable suture material, insulin, and microchips have also been linked with its development [14]. Hence, inflammation, regardless of its origin, appears to facilitate the development of FISS. While it has been suggested that FISS differs from the non-injection associated FSA based on histopathological features [15,16], the criteria to distinguish between the two conditions cannot be considered as conclusive [17]. Hence, it remains unknown whether and how the different manifestations of FSA differ on a molecular level.

The current gold standard for treatment of FSA in all species is a complete surgical excision. However, distinguishing tumor from unaffected normal tissue (NT) poses significant challenges due to highly infiltrative growth that requires large surgical margins to ensure clean resections [11,14]. This is especially true in cats, as studies have repeatedly demonstrated that the first cut is the best chance for cure. Due to the invasive nature of the disease, the current recommendation for surgical margins in cats is 5 cm lateral and two fascial planes deep, representing the most aggressive surgical dose in sarcoma resection across species [[18], [19], [20]]. Incomplete resections cause tumor relapse and a significantly negative impact on prognosis. Additional neo-/adjuvant radio- and chemotherapy protocols offer limited success [21,22]. Therapeutic outcome for FSA in all species could greatly benefit from targeted modalities, such as precise tumor visualization using targeted dyes to improve resection [23] or targeted delivery of cytotoxic payloads, particularly in the metastatic setting. However, development of such targeted modalities is hindered by the lack of molecular data identifying targets that differentiate FSA from unaffected NT.

While feline and adult FSA share histomorphologic traits and exhibit similar clinical behavior, there is a striking lack of detailed molecular characterization for both entities. Thus, it remains unclear how feline and adult FSA compare on a molecular level. Moreover, it remains obscure what differentiates tumor from healthy surrounding NT in either species, preventing identification of tumor-specific targets that could be leveraged in the context of targeted therapy or targeted visualization strategies to improve patient outcome. As such, the shortage of data on both molecular cross-species homology and the differences between tumor and NT impedes the translational potential of feline FSA for development of novel therapeutic approaches that could greatly benefit human patients.

We have established a powerful approach to profile transcriptomic and proteomic changes in spatially defined areas of archival formalin-fixed paraffin embedded (FFPE) patient tumors using LCM followed by RNAseq and LC-MS/MS [[24], [25], [26], [27], [28], [29], [30], [31], [32], [33], [34]]. Here, we apply this approach to profile 30 cases of feline FSA and matched unaffected NT to gain detailed insight into the molecular changes and therapeutic vulnerabilities in spontaneous feline FSA.

## Material and methods

### Ethics approval and consent to participate

No animals were killed for the purpose of this research project, as the analyzed tissue had been surgically removed for curative reasons with the consent of the patient owners. According to the Swiss Animal Welfare Law Art. 3 c, Abs. 4 the preparation of tissues in the context of agricultural production, diagnostic or curative operations on the animal or for determining the health status of animal populations is not considered an animal experiment and, thus, does not require an animal experimentation license. The use of FFPE material from feline patients which was obtained for diagnostic reasons therefore does not require a formal ethics approval and complies with national guidelines. The project was subjected to an institutional ethics review and approved by the Ethics Committee of the Faculty of Medicine, University of Zurich (MeF-Ethik-2024-01).

### Selection of cases for LCM

Fibrosarcoma and matched unaffected NT (skeletal muscle (SM), adipose tissue (AT) and connective tissue (CT)) were concurrently isolated using laser-capture microdissection from FFPE tissue of 30 feline FSA samples that were provided by the Institute of Veterinary Pathology of the Vetsuisse Faculty Zurich. Based on the clinical history and the anatomic location, an injection-related origin (i.e. FISS) cannot be ruled out for any of the cases (i.e. all patients have received vaccinations). However, in the absence of definite markers to differentiate between FISS and non-FISS FSA, and the unresolved question whether the two subtypes really differ on a molecular level, all tumors were considered as ‘FSA’. All samples were either from the Small Animal Hospital of Zurich or external cases sent in by veterinarians practicing in Switzerland. Cases were reviewed and selected by a certified pathologist (FG) according to the criteria indicated by [35]. Paraffin blocks were routinely kept at room temperature. Tissue processing for LCM was performed as previously described [25]. Table 1 provides clinical details for all cases included in the study.

**Table 1:** Overview of FSA cases included in the study. ESH = European short hair. Age = age of patient at excision of tumor. f = female, m= male, n = neutered. Anatomical location as indicated in medical records. SM/AT/CT indicate which matched normal tissues were collected for the respective cases, where AT = Adipose tissue, CT = Connective tissue, SM = Skeletal muscle. Outcome group indicates the cases that were assigned to the highly aggressive (HA) or low-aggressive (LA) groups, respectively.

Case ID	Breed	Age	Sex	Anatomical location*	SM	AT	CT	Outcome group
T-5	ESH	15	f/n	Skin abdomen	x	x	x	
T-6	ESH	11	f/n	Skin flank	x	x	x	
T-7	ESH	17	m	Skin back	x	x	x	HA
T-8	ESH	15	f	Skin chest	x	x	x	HA
T-9	ESH	14	m	Skin back		x	x	HA
T-10	ESH	15	m	Skin flank	x	x		HA
T-11	ESH	12	m	Skin flank	x	x	x	
T-12	ESH	12	m	Skin flank	x	x	x	
T-13	ESH	10	m	Skin shoulder	x	x	x	LA
T-14	ESH	16	m	Skin hindlimb			x	LA
T-15	ESH	13	m/n	Skin hindlimb		x		LA
T-16	ESH	15	f/n	Skin flank		x		
T-17	ESH	12	m	Skin back		x	x	
T-18	Maine Coon	11	m/n	Skin abdomen	x	x		LA
T-19	ESH	13	f	Skin back	x	x	x	
T-20	ESH	8	f/n	Skin chest	x	x	x	
T-21	ESH	12	f/n	Skin shoulder		x	x	
T-22	ESH	10	m	Skin shoulder	x	x	x	
T-23	Persian Mix	9	f	Skin back	x	x		
T-24	Maine Coon	12	m	Skin chest	x	x	x	LA
T-25	ESH	17	f/n	Skin back		x	x	LA
T-26	ESH	13	f	Skin thigh		x	x	
T-27	ESH	10	f	Skin neck		x	x	LA
T-28	ESH	5	f/n	Skin back		x	x	
T-29	ESH	13	f	Skin shoulder	x	x	x	
T-30	ESH	12	f	Skin shoulder	x		x	
T-31	ESH	14	f	Skin shoulder	x	x	x	HA
T-32	ESH	12	f	Skin shoulder	x		x	
T-33	ESH	8	f	Skin abdomen	x	x	x	
T-34	ESH	15	f	Skin chest	x	x	x	

### Laser-capture microdissection (LCM)

Laser-capture microdissection was performed using the ArcturusXT^TM^ Laser Capture Microdissection System (Thermo Scientific) as described in [25]. Areas of interest identified by a certified pathologist (FG) were isolated according to the manufacturer's protocol and the criteria described in [32]. Selectivity of isolation was verified by microscopic examination of the LCM cap as well as the excised region after microdissection. 2 caps were collected per case and tissue. After excision, the thermoplastic membranes containing captured tissue were peeled off the caps using a sterile scalpel and forceps and subsequently stored in a 1.5 ml centrifuge tube (EppendorfⓇSafe-Lock tubes) and frozen at −20°C until further processing.

### Sample preparation for proteomic analysis

For proteomic analysis, all samples were processed in a single batch. For protein extraction, sterile blades and forceps were used to peel off the thermoplastic membranes containing captured cells from the cap, which were then transferred into a sterile Eppendorf® Safe-Lock tube. Microdissected tissue was rehydrated by adding 900 μl of heptane and incubating for 10 min at 30°C in a thermomixer (800 rpm). After centrifugation (20′000 x *g*, 10 min), the heptane was removed, and the step was repeated. Subsequently, the membranes were washed with 900 μl of ethanol (5 min, RT, 1′000 rpm), 200 μl of 90 % ethanol (5 min, RT, 1′000 rpm) and 200 μl of 75 % ethanol (5 min, RT, 1′000 rpm). The samples were stored at -80°C overnight. The samples were then prepared using a commercial iST Kit (Pre-Omics, Germany) with an updated version of the protocol, as described in [32].

### Liquid chromatography-mass spectrometry analysis

LC-MS/MS analysis was performed on an Orbitrap Fusion Lumos (Thermo Scientific) equipped with a Digital PicoView source (New Objective) and coupled to an M-Class UPLC (Waters). Solvent composition of the two channels was 0.1 % formic acid for channel A and 99.9 % acetonitrile in 0.1 % formic acid for channel B. Column temperature was 50°C. For each sample 3 µl of peptides were loaded on a commercial ACQUITY UPLC M-Class Symmetry C18 Trap Column (100Å, 5 µm, 180 µm x 20 mm, Waters) connected to a ACQUITY UPLC M-Class HSS T3 Column (100Å, 1.8 µm, 75 µm X 250 mm, Waters). The peptides were eluted at a flow rate of 300 nl/min. After a 3 min initial hold at 5 % B, a gradient from 5 to 24 % B in 80 min and 22 to 36 % B in additional 10 min was applied. The column was cleaned after the run by increasing to 95 % B and holding 95 % B for 10 min prior to re-establishing loading condition. Samples were measured in randomized order. For the analysis of the individual samples, the mass spectrometer was operated in data-independent mode (DIA). DIA scans covered a range from 400 to 1000 m/z in windows of 16 m/z. The resolution of the DIA windows was set to 30′000, with an AGC target value of 500′000, the maximum injection time set to 50 ms and a fixed normalized collision energy (NCE) of 33 %. Each instrument cycle was completed by a full MS scan monitoring 350 to 1500 m/z at a resolution of 120′000. The mass spectrometry proteomics data were handled using the local laboratory information management system (LIMS) [36].

### LC-MS/MS data processing

The acquired MS raw data were processed for identification and quantification using FragPipe (version 19.0), MSFragger (version 3.6), and Philosopher (version 4.8.1) [37]. Spectra were searched against a Uniprot Felis catus database (taxonomy ID 9685, downloaded on 11.05.2023) concatenated to its reversed decoy database, and common protein contaminants. MSFragger-DIA mode for direct identification of peptides from DIA data was used. Strict trypsin digestion with a maximum of two missed cleavages was set. Carbamidomethylation of cysteine was selected as a fixed modification, while methionine oxidation was set as variable modifications. EasyPQP was used to generate a DIA-NN-compatible spectral library. Subsequent quantification was performed with DIA-NN version 1.8.2.

### LC-MS/MS data analysis

Differential protein expression analysis was performed using the r-package prolfqua [38]. The intensities were first log_2_ transformed and then z-transformed so that the sample mean and variance were equal. Next, we fitted a linear model with a single factor (tissue) to each protein, and tissue differences (protein log_2_ fold changes (log_2_(FC)) were estimated and tested using the model parameters. To increase the statistical power, the variance estimates were moderated using the empirical Bayes approach, which exploits the parallel structure of the high throughput experiment [39]. Finally, the p-values are adjusted using the Benjamini and Hochberg procedure to obtain the false discovery rate (FDR).

### RNA isolation and sequencing

RNA was isolated using the Covaris® truXTRAC FFPE RNA kit and the Covaris® E220 focused ultrasonicator as established [[25], [26], [27], [28],^30^,^31^]. RNA abundance and quality was analyzed using the 4200 or 2200 Tape Station Software using the High Sensitivity RNA ScreenTape kit (Agilent Technologies), according to the manufacturer's protocol. For RNAseq, all samples were processed in a single batch. 10 ng of RNA diluted to a concentration of 0.33 ng/μl in a total volume of 30 μl was submitted for RNA sequencing, as detailed in [26]. The SMARTer Stranded Total RNAseq Kit-Pico Input Mammalian (Clontech/Takara Bio USA) was used according to the manufacturer's protocol for RNA library preparation and ribosomal RNA depletion. Single-read sequencing (125 bp) was performed in a single batch for all samples of the same tumor type and species using the Illumina HiSeq 4000 according to standard protocols of the Functional Genomics Centre Zurich (FGCZ).

### RNAseq data processing

The raw reads were cleaned by removing adapter sequences, trimming low-quality ends, and filtering reads with low quality (phred quality < 20) using Trimmomatic (version 0.36) [40]. Sequence pseudoalignment of the resulting high-quality reads to the feline reference genome Felis_catus_9.0, Release_102-2021-02-02, and quantification of gene-level expression was carried out using Kallisto (version 0.44) [41]. Gene counts were imported into the R/Bioconductor package EdgeR [42] (R, version 3.6.1, EdgeR, version 3.28), and trimmed mean of M values normalization size factors were calculated to adjust for sample differences in library size. The generalized linear model was used to detect differentially expressed genes incorporating adjusted (Benjamini and Hochberg method) p-values.

### Pie chart

Pie charts were plotted using the PieChart function from the lessR R package [43].

### Venn diagram

Venn diagrams were produced either using ggvenn R package [44] or the BioVenn R package [45] for proportional diagrams. Identification of tumor and NT-specific proteins was performed by separating data according to tissue group and filtering by row mean !=0 to ensure presence in at least one sample. The intersection of each tissue group was used to calculate overlapping proteins and separate tissue-specific targets. Furthermore, the intersection of differentially expressed genes and proteins between tumor and the different NT was used to identify common differential expressed genes and proteins.

### Heatmap

Heatmaps were generated using R package ComplexHeatmap [46] with row clustering distance was set to “Euclidean” and RowAnnotation according to overall high, mid and low expression. HALLMARK and pathway analysis of high and lowly expressed proteins was performed with molecular signatures database (MSigDB) [47].

### Barcodeplot

Cross-species comparative analysis of tumor-specific expression was performed using the barcode enrichment plot from limma [48]. The proteomic dataset by Tang *et al,* 2024 [49] was used as external human dataset, and the canine data was from [32]. All target identifiers from the external datasets were summarized at the gene level using BioMart [50]. Raw data was log_2_ normalized and genes were ranked according to their mean expression across all samples. Plots show the ranked position indicating the expression in the feline cohort (x-axis) compared to the ranked expression in the external human and canine datasets (line extension of the y-axis). Only common genes in feline, canine and human were included in the barcodeplot analysis. Pearson correlation analysis of ranked position was used to confirm significance. The top 100 common highly expressed genes from each plot were identified as the leading edge and selected for input in the Venn diagram.

### Gene set enrichment analysis (GSEA) and over representation analysis (ORA)

For GSEA, ORA and KEGG pathway analysis, the tool WebGestalt (http://www.webgestalt.org) [51] or the molecular signatures database (MSigDB v2023.2.Hs) [52,53] were used. Additional pathway analysis was performed with the help of QIAGEN Ingenuity Pathway Analysis (QIAGEN Inc., https://digitalinsights.qiagen.com/IPA) comparing the differentially expressed proteins in tumor to each NT.

### Proteomic and genomic data integration

In addition to gene set enrichment analysis, we also performed ssGSEA using the public server from GenePattern [54] (https://www.genepattern.org/#gsc.tab=0) to calculate separate enrichment scores for each pairing of a gene set and tumour sample. Principle component analysis was performed applying prcomp on normalized protein intensity values. 2D and 3D visualization was achieved using R packages ggplot2 [55] and scatterplot3d [56], with the first two or three principal components as x, y and z axis values respectively. For the comparison of the transcriptomic and proteomic data set, the online tool Shiny App (https://fgcz-shiny.uzh.ch/connect/) run by the Functional Genomics Center Zurich, was used. Uniprot protein identifiers were first converted to ensembl and then gene names. Feline genes (Felis_catus_9.0) were converted to human orthologues using Ensembl BioMart (release 100) prior to analysis with MetaCore [57]. For the pathway analysis, the web tool MetaCore from Clarivate AnalyticsTM was used (https://portal.genego.com).

### Survival analysis

Association of gene expression with disease-free interval or overall survival in the human TCGA-SARC dataset (http://cancergenome.nih.gov/) [58] was performed using GEPIA 2.0 [59] (http://gepia.cancer-pku.cn).

### Cell culture

FSII and FSIII cells were a kind donation from Prof. M. Reinacher (Department of Veterinary Pathology, Justus-Liebig-University of Giessen, Germany) [60]. Cells were cultured under standard conditions @ 37°C in humid atmosphere with 5 % CO_2_ in Gibco™ DMEM, low glucose, GlutaMAX™ Supplement with 15 % FCS (Gibco), MEM-Nonessential amino acids (Gibco) and antibiotic-antimycotic supplement (Gibco), and regularly tested for mycoplasma. Twenty-four hours before treatment, 2,500 cells were seeded in 100 μl complete medium into 96 well plates. Drugs used were: Vincristin (Teva Pharma AG), Vinorelbine (Sandoz Pharmaceuticals AG), Vinblastin Sulfate (Teva Pharma AG), Cytarabine (Pfizer Switzerland), Actinomycin D (Sigma), Carboplatin (Accord Healthcare AG), Doxorubicin (Teva Pharma AG), Gemcitabine (Fresenius), ATMi (KU-55933, Sigma), ATRi (AZ-20, Selleck Chemicals), and PARPi (Olaparib, Selleck Chemicals). For all experiments, stock solutions of inhibitors were serially diluted in complete medium to obtain the required concentrations and used to replace the seeding medium. Medium was replaced after 96 hours in experiments lasting 6 days. After the incubation period, medium was replaced with fresh medium containing 0.025 mg/ml Resazurin in PBS, and plates were further incubated at 37°C. Sample fluorescence was measured after 2 to 4 hours incubation using the BioTek Synergy H1 Plate Reader (Agilent Technologies) set to ex = 560 and em = 590. Mean values of 4 to 6 replicate wells were calculated for each treatment point and cell line and normalized to control treated cells.

### Graphical display of results

GraphPad Prism, Shiny App and MetaCore were used for calculation of IC50 and visual representation of the results, along with selected R packages previously mentioned.

## Results

### Spatially defined multiomic profiling of tumor and matched NT in a cohort of 30 feline FSA

**Fig. 1:**
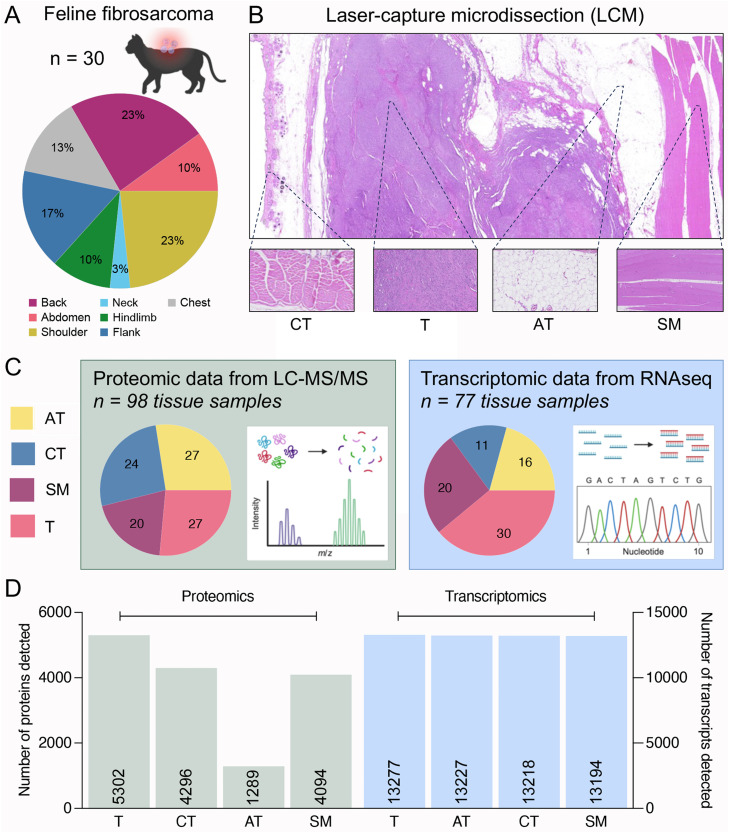
Overview of proteins and transcripts identified in feline fibrosarcoma and surrounding peritumoral tissue of 30 cases. A) Pie chart illustrating the anatomical locations of the patients’ tumors. B) Experimental approach to isolate FSA, adipose tissue (AT), connective tissue (CT) and skeletal muscle (SM) from FFPE sections using LCM, followed by LC-MS/MS and RNAseq. C) Number of samples per tissue type analyzed using LC-MS/MS or RNAseq. D) An overview of the total count of identified proteins and transcripts across all samples and tissue types. Parts in panels A and C were created with BioRender.com.

The cohort of feline FSA is composed of 30 primary tumors from 27 European Shorthair, two Maine Coon and one Persian mix breed cats, of which 17 were female (8 neutered) and 13 male (2 neutered) (Table 1). Median age was 12 years, and anatomical sites affected (as per medical reports) included abdomen, back, chest, flank, hindlimb, neck, shoulder, and thigh (Fig 1A). Based on the clinical history (vaccinations) and the anatomic location, an injection-related origin (i.e. FISS) cannot be ruled out for any of the cases. However, in the absence of definite markers to differentiate between FISS and non-FISS FSA, all tumors were considered as ‘FSA’.

To gain insight into the proteomic and transcriptomic landscape of these tumors, we applied LCM to isolate tumor and matched unaffected connective tissue (CT), adipose tissue (AT) and skeletal muscle (SM) from all cases in this cohort (Fig. 1B). All the matched normal tissues (NT) are frequently found in the vicinity of FSA and hence present tissue that the tumor needs to be differentiated from for targeting purposes in a clinical setting. Subsequently, LCM-isolated samples were analyzed by LC-MS/MS and RNAseq, respectively. The final sample set analyzed by LC-MS/MS consisted of a total of 98 tissue samples (27 tumor, 27 AT, 24 CT and 20 SM), while the final RNAseq dataset was composed of 77 specimens (30 tumor, 16 AT, 11 CT and 20 SM) (Fig. 1C). In total, proteomic analysis detected 5′302 different proteins in all tumor samples, 4′296 in CT, 1′289 in AT and 4′094 in SM, with an average of 4554 proteins detected per tumor sample, 2′139 in CT, 489 in AT and 2′468 in SM (Fig. 1D, Supplementary Tables 1 and 2, and Supplementary Fig. 1). Of these, 2′324 proteins were commonly detected in every T, 389 in CT, 97 in AT and 1′197 in SM. Transcriptomic analysis identified a total of 13277 transcripts across all tumors, 13′277 in CT, 13′218 in AT and 13′194 in SM (Fig. 1D, Supplementary Table 3). 7′454 transcripts were shared across every tumor sample, 6′251 in CT, 6′464 in AT and 5′022 in SM. As such, this represents the first detailed proteomic and transcriptomic dataset of feline FSA and its surrounding NT.

### Transcriptomic profiling of feline FSA identifies transcripts highly overexpressed in tumor compared to unaffected NT

**Fig. 2:**
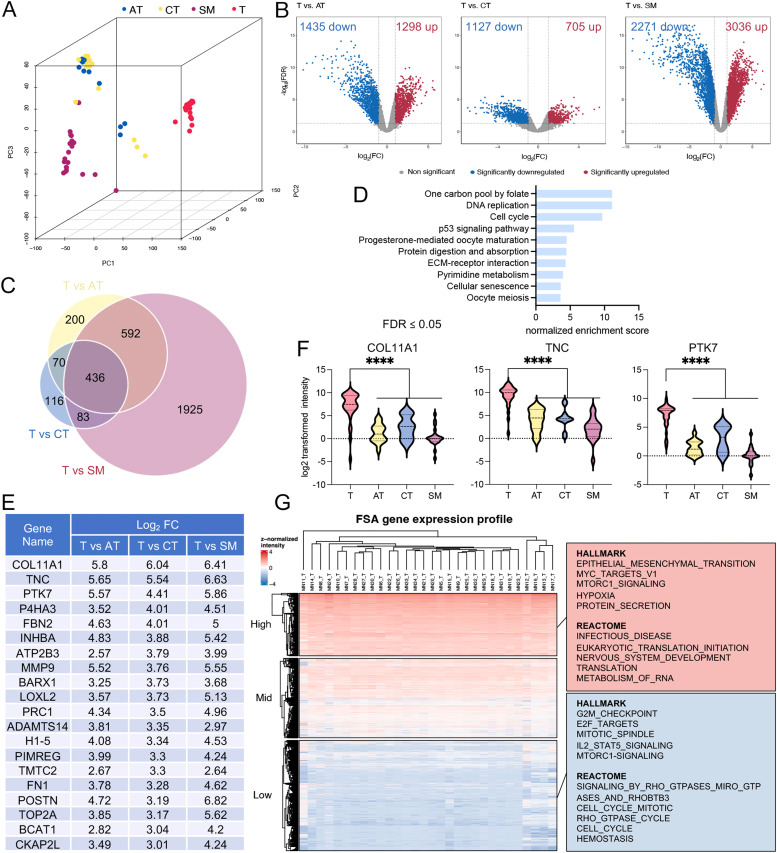
Transcriptomic analysis of FSA and matched NT highlights unique expression patterns of tumors compared to the surrounding tissue. A) 3D-Principal Component Analysis plot of genes detected in tumor and each NT from 30 feline FSA. PCA was performed using all identified genes. B) Volcano plots featuring DEG between the tumor and each NT. Cutoff values for significance (|log_2_FC| ≥ 1 and FDR < 0.05) are indicated using grey dashed lines. P-values were calculated using ANOVA. C) Venn diagram illustrates all upregulated genes in the tumor compared to each NT, using a threshold of log_2_FC of ≥ 1 and FDR < 0.05. D) ORA analysis using KEGG pathway of the 436 commonly upregulated genes in tumor. E) Top 20 most differentially expressed genes in tumor compared to each NT (log_2_FC ≥ 1, FDR < 0.05). F) Violin plot of selected transcripts from E to illustrate expression in tumor and adjacent NT (****P < 0.0001, calculated using ANOVA). The dashed lines indicate the interquartile range and the dotted line represents the median. G) (left) Heatmap of all T samples using all transcripts. (right) GSEA using HALLMARK and REACTOME pathway databases of highly (top) and lowly (bottom) expressed RNAs in FSA. T = tumor, AT = adipose tissue, CT = connective tissue, SM = skeletal muscle.

Principal component analysis (PCA) using all identified transcripts clearly separated tumor from the different normal tissue types within the first three principal components (Fig. 2A). Of note, the overlap between AT and CT was presumably due to AT having low RNA contents in general and the presence of fibroblasts as a structural feature in AT, which contributes a CT-like expression signature. This supported the validity of our approach to analyze spatially defined tissue regions using RNAseq and highlighted the difference between the tissue types as the major source of variability, overriding any potential effects due to differences in breed, anatomical location of the tumor or other clinical features. Analysis of differentially expressed genes (DEGs) between tumor and each normal tissue (cut-off values for significance: log_2_(FC) > 1 and < -1, FDR < 0.05) identified 1′163 significantly up- and 1′331 significantly downregulated targets between tumor and AT, 638 up- and 1′102 downregulated between T and CT and 2′736 up- and 2′072 downregulated in T vs SM (Fig. 2B).

Gene set enrichment analysis (GSEA) of expression changes using the KEGG database revealed a strong enrichment of pathways related to cell cycle, DNA replication and repair and RNA production in tumor tissue compared to all three NTs separately (Supplementary Fig. 2). In contrast, AT was characterized by pathways involved in lipolysis, AMPK, PPAR, and Adipocytokine signaling, while CT featured cytokine-cytokine receptor interactions, cell adhesion molecules and complement cascade. SM was characterized by typical muscle-related pathways, including muscle contraction, adrenergic and insulin signaling, confirming the specificity of tissue isolation (Supplementary Fig. 2). Reactome pathway analysis further supported these findings (Supplementary Fig. 3). Of the transcripts significantly upregulated in tumor, 436 targets were commonly upregulated by a log_2_(FC) > 1 across all three individual comparisons, representing potential candidates for markers that discriminate tumor from all NTs (Fig. 2C and Supplementary Table 4). Overrepresentation analysis of these 436 targets using KEGG pathway analysis revealed involvement in one carbon metabolism, DNA replication, cell cycle and p53 signaling, among others (Fig. 2D). The top 20 targets highly upregulated in tumor compared to all NT (ranked according to the log_2_(FC) T vs CT) include transcripts encoding for COL11A1, TNC, PTK7, and P4HA3 (Fig. 2E and F). Unsupervised hierarchical clustering of tumor tissue alone revealed a somewhat heterogeneous structure, suggesting several subclusters within the data (Fig. 2G). GSEA with the HALLMARK and Reactome databases revealed an enrichment of epithelial to mesenchymal transition, Myc targets, mTORC1 signaling, translation, infectious disease and nervous system development among the highly expressed genes. In contrast, the lowly expressed genes were enriched for pathways including G2M checkpoint, E2F targets, and cell cycle signaling events (Fig. 2G). In summary, feline FSA display a distinct transcriptional profile strongly dominated by pathways centered around the cell cycle, DNA repair and DNA replication that clearly differentiates them from unaffected NT.

### Proteomic profiling of feline FSA and matched NT reveals potential tumor-specific markers

**Fig. 3:**
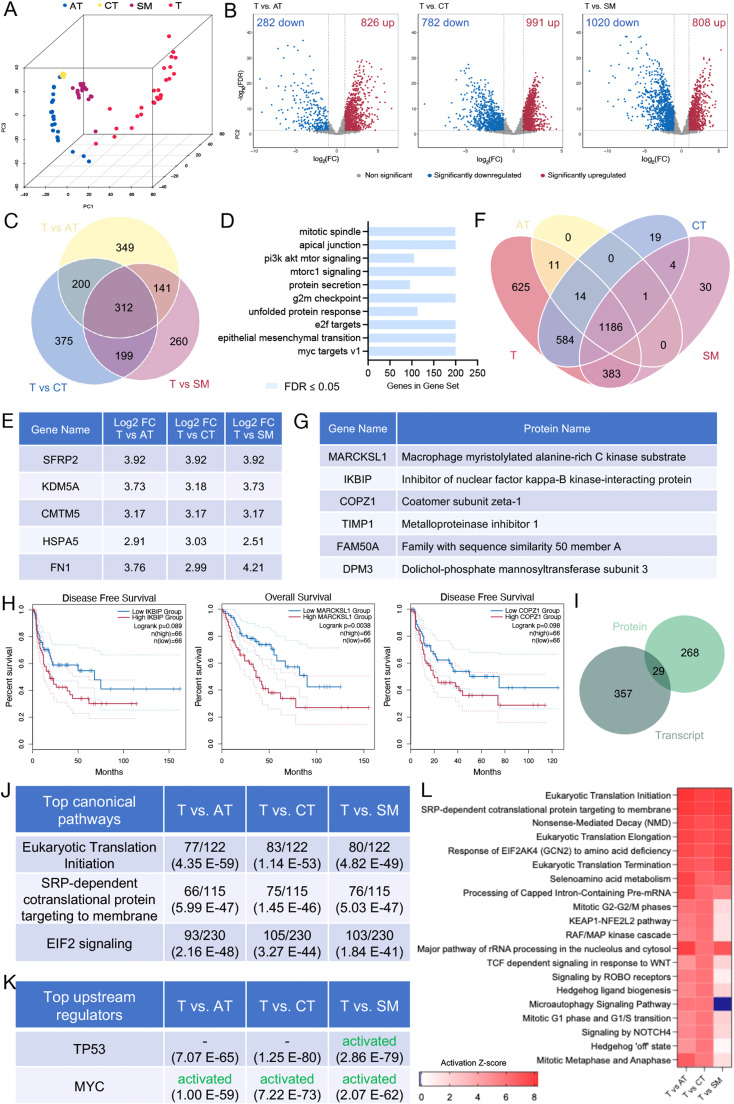
Proteomic analysis of FSA and its NT highlights unique expression patterns of tumor compared to the surrounding. A) 3D-Principal Component Analysis plot of proteins detected in tumor and each NT from 27 feline FSA. PCA was performed using all identified proteins. B) Volcano plots featuring differentially expressed proteins between the tumor and each NT. Cutoff values for significance (|log_2_FC| ≥ 1 and FDR < 0.05) are indicated using grey dashed lines. P-values were calculated using ANOVA. C) Venn diagram illustrates all upregulated proteins in the tumor compared to each NT, identified with a threshold of log_2_FC of > 1 and FDR < 0.05. D) GSEA using HALLMARK pathways on all commonly upregulated proteins in tumor compared to NT. E) Top 5 most abundant proteins detected in T compared to each NT (log_2_FC > 1, FDR < 0.05). F) Venn diagram showing proteins identified across all tissue types. G) List of the 6 tumor-exclusive proteins that were detected in every single tumor sample. H) Kaplan-Meyer curves for high/low IKBIP, MARCKSL1 and COPZ1 expressing tumors using the TCGA-SARC dataset. I) Venn diagram of the overlap of proteins and transcripts significantly upregulated in T vs all NT. J) Top canonical pathways were detected in T vs AT, CT and SM using ingenuity pathway analysis (IPA). Values on the top indicate the number of detected targets / number of targets attributed to the respective dataset; values in brackets represent p-values. K) Top upstream regulators detected in T vs AT, CT and SM using IPA. The top indicates the predicted activation status, and the values in brackets represent p-values. L) The activation status of the top 20 upregulated canonical pathways in T vs AT, CT and SM.

Similarly to the RNA data, PCA differentiated between the four tissue types within the first three principal components on the protein level (Fig. 3A). Analysis of differentially expressed proteins between tumor and each NT (cut-off values for significance: log_2_(FC) > 1 and < -1, FDR < 0.05) identified 826 significantly up- and 282 significantly downregulated proteins between tumor and AT, 992 up- and 782 downregulated proteins between T and CT and 808 up- and 1′020 downregulated proteins in T vs SM (Fig. 3B). GSEA of expression changes using KEGG pathways between tumor and the NTs identified pathways involved in ribosome or protein assembly, protein processing in the endoplasmic reticulum and antigen processing and presentation as positively enriched in tumor tissue (Supplementary Fig. 4). Of the proteins detected as significantly upregulated in tumor, 312 were shared across all three individual comparisons, representing potential tumor-specific targets (Fig. 3C and Supplementary Table 5). GSEA of these targets using HALLMARK revealed involvement in PI3K-Akt-mTor signaling, G2M checkpoint, Myc targets and epithelial to mesenchymal transition, among others (Fig. 3D). The top 5 overexpressed proteins in tumor compared to NT (ranked according to the log_2_(FC) T vs CT) included SFRP2, KDM5A, CMTM5, HSPA5 and FN1 (Fig. 3E).

Of all detected proteins, 19 were found only in samples of CT, 30 were specific to SM, and 625 were exclusive to tumor tissue, while none were detected only in AT (Fig. 3F and Supplementary Table 6). ORA using HALLMARK pathway of these 625 tumor exclusive proteins identified enrichment of Mitotic spindle, E2F targets, G2M checkpoint, inflammatory response, and Myc targets among others (Supplementary Fig. 5). 137 of these proteins were detected in >80 % of cases (i.e. 22/27), 77 in 90 % (i.e. 24/27) and 6 proteins were present in every single tumor sample analyzed (Supplementary Table 7). These 6 tumor-exclusive proteins detected in every single sample comprised MARCKSL1, IKBIP, COPZ1, TIMP1, FAM50A and DPM3 (Fig. 3G).

As feline FSA are considered highly malignant forms of STS, we next evaluated whether these tumor-exclusive proteins were associated with tumor aggressiveness. To this end, we assessed the association of their expression with disease-free interval or overall survival in human STS using the TCGA-SARC dataset (Fig. 3H). Indeed, this analysis found high levels of IKBIP, MARCKSL1 and COPZ1 levels to be associated with shorter disease-free interval or survival (IKBIP: disease-free interval (p = 0.089), MARCKSL1: overall survival (p = 0.0038), COPZ1: disease-free survival (p = 0.098)). Therefore, these data suggest that high expression of these proteins is associated with a negative impact on human STS.

It is well-established that increased levels of RNA do not necessarily translate to increased protein levels. Correlation between proteomic and transcriptomic data using the log_2_(FC) values from comparisons of T vs AT, CT, and SM were very moderate (r = 0.37 for AT, r = 0.37 for CT, and r = 0.53 for SM; Supplementary Fig. 6). As such, this demonstrates that transcriptomic and proteomic analysis of patient tissue yields complementary information and enables a more comprehensive view than either analysis alone. To understand which of the significantly upregulated proteins in tumor were also upregulated on the RNA level, we computed the overlap between the datasets. The Venn diagram revealed an overlap of 29 shared targets, including FN1, POSTN, and RUNX2, further validating the upregulation of these targets in feline FSA (Fig. 3I and Supplementary Table 8).

Analysis of the expression differences between tumor and NT using QIAGEN Ingenuity Pathway Analysis detected eukaryotic translation initiation, SRP-dependent co-translational protein targeting to membrane and EIF2 signaling as the top canonical pathways in all three comparisons of tumor vs NT (Fig. 3J). Identification of top upstream regulators revealed TP53 and MYC activation in tumor tissue (Fig. 3K). Finally, assessment of the activation status of the top 20 activated canonical pathways further reinforced the massive emphasis on RNA- and translation-related pathways in feline FSA, as well as involvement of WNT and hedgehog signaling (Fig. 3L).

In conclusion, the proteomic signature clearly differentiates feline FSA tumor tissue from unaffected AT, CT and SM, revealing a massive dependence on translation-related pathways and a significant number of proteins either strongly overexpressed in or restricted to tumor tissue that could potentially serve as tumor-specific markers.

### Feline FSA comprises subtypes characterized by neuronal, fibroblastic and inflammatory expression patterns potentially associated with differences in clinical behavior

**Fig. 4:**
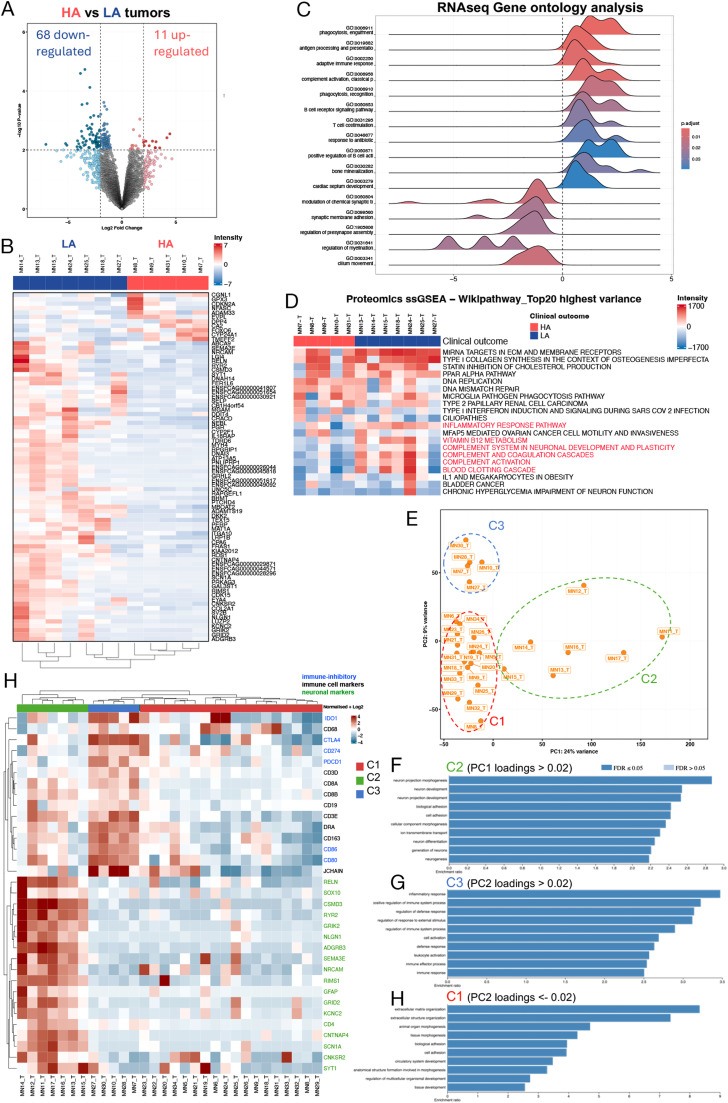
Identification of transcriptomic and proteomic features in feline FSA associated with differences in clinical behavior. A) Volcano plot of differentially expressed genes between highly aggressive (HA) and lowly aggressive (LA) tumors. Cutoff values for significance: |log_2_FC| ≥ 2 and p = 0.01. B) Heatmap showing unsupervised clustering of significant differentially expressed genes from A. C) Ridge plot showing Gene Set enrichment analysis of differentially expressed genes from B. D) Top 20 pathways with the highest variance between HA and LA tumors in the proteomic dataset assessed using single sample GSEA and Wikipathway. E) PCA of all tumors using all transcripts with the three molecular clusters C1 (fibroblastic), C2 (neuronal-like) and C3 (inflammatory) indicated in dashed lines. F-H) ORA of PC1 loadings > 0.02 (F), PC2 loadings > 0.02 (G), or PC2 loadings <-0.02 (H) from E using Gene Ontology of Biological Processes. I) Heatmap of unsupervised clustering results, based on transcripts restricted to neuronal and immune-related features, differentiates between the three clusters C1 – C3.

Assessment of patient outcome within the cohort allowed identification of two subgroups of patients with differing clinical outcome. Five patients that showed worse survival time than expected (i.e. survival < 500 days when excised with clean margins or < 60 days with unclean margins or cases with metastatic disease/systemic failure) were classified as ‘highly aggressive’ (HA), while 7 patients that surpassed survival > 500 days after resection (some even despite R1 margins) and without metastases were classified as ‘low-aggressive’ (LA; Table 1 and Supplementary Table 9). Differential gene expression analysis between these two groups using log_2_(FC) > 2, p < 0.01 detected 79 significantly deregulated targets (11 up- and 69 downregulated), which also clearly separated both groups by unsupervised clustering (Fig. 4A and B). GSEA revealed a strong enrichment of immune-related pathways including phagocytosis, engulfment, antigen processing and presentation, adaptive immune response, and B cell receptor signaling in the HA group (Fig. 4C). In contrast, processes involved in transmembrane transport, synaptic membrane adhesion, regulation of presynapse assembly and myelination as well as cilium movement were enriched in LA tumors, potentially hinting at a more neuronal-like differentiation state of these tumors (Fig. 4C). To assess functional differences between these two groups on the protein level, we next investigated the respective LC-MS/MS data using single sample Gene Set Enrichment Analysis (ssGSEA) focusing on the top 20 pathways with highest variance. Interestingly, this identified clear differences in the inflammatory response pathway as well as the complement and coagulation cascade, both of which were highly enriched in LA tumors as opposed to HA tumors (Fig. 4D). Moreover, the HA tumors were characterized by a significantly higher enrichment of DNA replication and DNA mismatch repair-associated pathways. These data suggest that differences in immune-related and neuronal RNA expression patterns are associated with tumor aggressiveness in feline FSA due to functional differences in the activation status of inflammatory response, complement and coagulation as well as DNA replication.

To further explore feline FSA in molecular detail, we expanded the analysis to all included cases. Interestingly, PCA of tumor samples using all transcripts suggested the existence of 3 different tumor clusters: while 17 samples grouped tightly together to form a main cluster (C1), a second cluster (C2) composed of 7 tumors was clearly separated along PC1 from the main bulk of specimens, and a third cluster of 5 tumors was separated along PC2 (C3) (Fig. 4E). To understand the molecular differences driving these three clusters, we further assessed the main loading factors driving PC1 and PC2. ORA of PC1 loadings using Gene Ontology of Biological Processes revealed highly significant enrichment of pathways involved in neuron development and differentiation as well as ion transmembrane transport (Fig. 4F), while PC2 loadings > 0.2 were enriched for processes involving inflammatory responses, the immune system and leukocyte activation (Fig. 4G). Finally, assessment of PC2 loadings < -0.02 revealed processes centered around extracellular matrix structure and organization, suggesting overrepresentation of mesenchymal functions associated with fibroblast function (Fig. 4G). As such, these results suggest the existence of three separate tumor subtypes within the morphological feline FSA cluster.

As the features that led to separation of C2 along PC1 were highly reminiscent of the neuronal features identified in the LA group, we manually curated the gene signature highly expressed in LA tumors to include only neuron-related targets (as per GSEA) and applied this to perform unsupervised hierarchical clustering of the full patient cohort. Strikingly, this revealed a clear separation of the samples in the C2 cluster from the other tumors (Fig. 4H). Hence, the tumors in cluster C2 seem to correspond to a neuronal-like STS subtype that is associated with less aggressive clinical behavior. Of note, these tumors showed expression of the two neuronal markers Sox10 and GFAP. Moreover, closer inspection of the main features driving the separation of C3 along PC2 identified the immunosuppressive factors IDO1, CTLA4 and CD80 among the top 12 loadings. Based on this, we assessed expression of these and other well-annotated immunosuppressive features (CD274/PD-L1, PDCD1/PD-1, CD86) as well as markers for T-cells (CD3, CD8A, CD8B) and B-cells (CD19, DRA, JCHAIN) (Fig. 4H). While T- and B-cell markers were present in both C2 and C3 tumor samples, C1 tumors appeared to be much less immune-infiltrated. Importantly, expression of immune-inhibitory molecules was strongly restricted to tumors of the C3 cluster, whereas the neuronal-like C2 tumors showed much less evidence of immunosuppression.

In conclusion, feline FSA comprise three different molecular subtypes characterized by neuronal-like, fibroblastic and inflammatory expression patterns that may influence clinical behavior. These results highlight the need for more refined molecular diagnostic approaches to improve classification and diagnosis of feline FSA.

### Feline FSA displays a high grade of molecular homology with both canine FSA and human fibroblastic sarcomas allows identification of tumor markers and therapeutic vulnerabilities

**Fig. 5:**
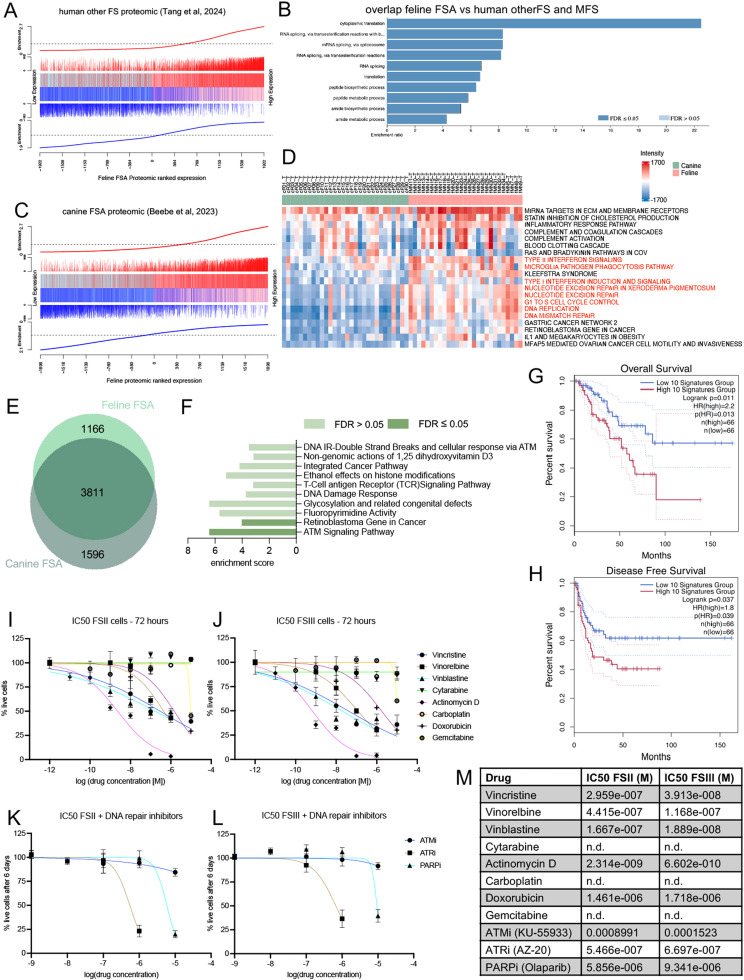
Cross-species assessment of feline, human and canine FSA. A) Competitive gene set testing to compare proteomic data from feline FSA and ‘human other FS’. B) ORA of the 199 shared highly expressed proteins between feline FSA, human other FS and human MFS. C) Competitive gene set testing to compare feline FSA proteomic data to canine FSA. D) Top 20 pathways from ssGSEA using Wikipathway for proteomic data from all canine and feline FSA. E) Venn diagram showing overlap of proteins identified in feline and canine FSA. F) ORA using KEGG pathways for the unique feline proteins in E. G – H) Kaplan-Meyer plot showing overall survival (G) and disease-free survival (H) in the TCGA-SARC cohort based on high/low expression of the 10-gene signature identified in F. I – L) Sensitivity of feline FSII (I, K) and FSIII (J, L) cells to various drugs after exposure for 72h (I, J) or 6 days (K, L). Data shown are mean from n = 6 assays ±SD. The colored lines indicate nonlinear fit IC50 curves for each cell line. M) Table detailing IC50 for both cell lines, as calculated from I – L.

Given the suggested similarity between feline, canine and adult FSA and in view of feline FSA as potentially useful model for the human condition, we next wanted to exploit the degree of molecular similarity across these three species. Due to the lack of transcriptomic data on adult FSA, we assessed interspecies similarity on the protein level, taking advantage of a recent proteomic dataset comprising 8 cases of ‘other fibroblastic sarcomas’ (other FS), the only available dataset for adult FSA [49], which however only contains matched normal CT for two cases. We postulated that if feline and human tumors were to share a high level of molecular homology, protein expression in the feline and human datasets should exhibit a similar expression pattern. To test this hypothesis and compare the two species, we ranked all tumor-derived proteins based on their expression in the feline dataset from low to high and assessed the enrichment of targets from ‘other FS’ in this list. Strikingly, the 50 % highly expressed genes in human STS (red vertical bars) were strongly enriched towards highly expressed genes in felines (right side) and vice versa for the lowly expressed proteins, demonstrating a very high correlation and wide-ranging conservation in protein expression between the two species (Fig. 5A). To further understand the overlap in significantly overexpressed features in feline FSA with human other FS, we computed the overlap between these two datasets. Interestingly, 251 of the 297 feline overexpressed proteins (15 did not have an annotated gene name and hence could not be compared) were also detected in human other FS (Supplementary Table 10). Importantly, 207 of these proteins had an average expression level among the top 32 % of all expressed proteins in human tumors, suggesting that these might represent good markers to differentiate tumor from adjacent NT also in human patients. Cross-comparison with the data for 26 human MFS, which are also fibroblastic tumors, revealed a highly similar picture, with 200 common proteins that had an average expression level among the top 29 % expressed proteins. ORA of the 199 proteins shared across both comparisons revealed a massive enrichment for RNA processing and ribosome-related processes, suggesting that targeting transcription could represent a potential therapeutic vulnerability in these tumors (Fig. 5B). As such, our feline dataset serves to identify features significantly overexpressed in tumor compared to normal tissue that are shared across species, supporting stratification of potential tumor-markers in the human dataset.

To extend our cross-species analysis to also include canine FSA, we next compared our proteomic dataset to a canine FSA LC-MS/MS dataset that we had generated using the same approach [32]. Again, comparison between the feline and the canine protein datasets revealed a very significant enrichment of highly and lowly expressed genes, respectively (Fig. 5C), suggesting a strong conservation of protein expression between the two species. Despite the wide-ranging homology between feline and canine FSA, feline FSA generally display a more aggressive clinical behavior than the canine counterpart. To address whether we could identify molecular features driving this clinical observation, we performed ssGSEA using Wikipathway for all canine and feline proteomic tumor samples. This revealed striking differences between the two species: in contrast to canine FSA, feline tumor samples had a strong enrichment for type I and II interferon signaling, phagocytosis and transactions involving DNA replication and repair mechanisms, including nucleotide excision repair and DNA mismatch repair (Fig. 5D). This suggested a tumor-promoting role for interferon-mediated immune transactions and DNA replication and -repair related features. To further assess interspecies differences, we performed ORA analysis using KEGG pathways to compare proteins found uniquely in the feline but not the canine tumor samples (Fig. 5E). This revealed a significant overrepresentation of ATM signaling, a key component in DNA damage signaling and repair, as well as retinoblastoma gene activity (Fig. 5F). As DNA repair pathways have been identified as interesting potentially druggable targets in a subset of human STS and have sparked currently running clinical trials [61,62], we further wanted to assess the relationship of the respective genes with clinical outcome in the human TCGA dataset. To do this, we generated a gene signature containing the relevant genes and examined their association with overall survival and disease-free interval (Supplementary Table 11). Strikingly, compared to patients with low expression of the signature, patients with high expression of our feline DNA repair signature had a significantly shorter disease-free interval as well as overall survival (Fig. 5G and H). As such, these results suggest feline FSA to represent highly interesting models for clinical assessment of therapies for very aggressive forms of human STS.

Adjuvant systemic therapy to improve tumor control after surgical excision would be highly beneficial but is complicated by the high chemoresistance of feline FSA. We therefore set out to explore potential therapeutic vulnerabilities based on our molecular insights using two feline FSA cell lines (FSII and FSIII, Fig. 5I - M). Consistent with a strong dependence on RNA transcription, both cell lines displayed a striking sensitivity towards Actinomycin D, an inhibitor of RNA transcription, with IC50 concentrations in the low nanomolar range or below (Fig. 5I and J). Further, in accordance with strong overrepresentation of mitotic spindle and G2M checkpoint activation, cells were sensitive towards the Vinca-Alkaloids Vincristin, Vinblastin, and Vinorelbin that interfere with microtubule polymerization and therefore block mitotic cell division, as well as Doxorubicin, a DNA intercalating agent. In contrast, the nucleoside analogues Cytarabine and Gemcitabine, as well as Carboplatin (a DNA cross-linking agent) failed to exert any significant effect on the feline FSA cells. Based on the overrepresentation of DNA repair signaling pathways in feline FSA, we next assessed the influence of specific inhibitors of ATM (KU-55933, ATMi), ATR (AZ-20, ATRi) and PARP (Olaparib, PARPi). While cells did not display sensitivity to ATMi, both ATRi and PARPi were able to induce significant reduction in cell viability after 6 days of incubation (Fig. 5K - M). This identifies the potential for ATR and PARP inhibitors for the treatment of feline FSA patients.

As such, our comprehensive molecular characterization identifies feline FSA as interesting and clinically amenable models for aggressive human STS, and identify therapeutic vulnerabilities based on their molecular features.

## Discussion

Human adult FSA represent very rare STS that are diagnosed based on exclusion due to a lack of subtype-specific diagnostic markers [4]. Specific molecular data on adult FSA is still exceedingly scarce with a total of n = 8 patients that have been analyzed by proteomic profiling [49], and no available RNAseq data to date. This lack of detailed molecular data on these tumors and how they differ from adjacent normal tissue impedes identification of diagnostic and therapeutic targets to develop novel approaches for affected patients. Moreover, even if novel therapy approaches are identified, the scarcity of the disease makes clinical assessment in affected patients practically impossible, a situation that is further exacerbated by the absence of clinically relevant models. Here, feline FSA represent a potential solution to support clinical assessment of novel therapies to benefit patients of both species. However, with transcriptomic data from only n = 3 FISS patients available [63], the molecular fingerprint of feline FSA remains obscure. This precludes identification of novel therapeutic strategies as hinders unbiased cross-species comparisons to assess the values and limitations of the feline model to inform on therapies to benefit patients of both species. To address this gap of knowledge, we provide a detailed molecular landscape of 30 cases feline FSA and its matched NT using tissue-resolved multiomic profiling that allows identification of tumor-specific targets and detailed insight into the molecular underpinnings of these feline tumors to improve diagnosis, prognosis, and treatment strategies for both species.

While spatial RNA sequencing approaches have recently emerged as to deliver spatially resolved transcriptomic insight into patient tissue [64], discovery-based detection of proteins within the tissue context is only emerging, and most available approaches, such as imaging mass cytometry, rely on antibody-dependent detection of a small number of targets [65]. This limits protein detection to predefined targets for which high-quality antibodies exist and is not directly adaptable to other species given difficulties in epitope conservation that interfere with antibody-mediated detection. Our tissue-resolved approach that can be applied to archival material represents an ideal workflow to species-independent discovery-driven assessment of patient tissue for both RNA and protein, especially in the case of STS, where rarity and heterogeneity of the disease add additional challenges to molecular investigations. Both transcriptomic and proteomic analysis of patient tissue offer exciting tools to complement genetic data and support translational research. Moreover, the combined assessment can significantly contribute towards understanding the molecular mechanisms driving STS growth and progression, identifying novel biomarkers or therapy responses and identification of novel therapeutic targets. In addition, proteins represent the largest and most functional group of druggable targets, and transcript levels do not necessarily correlate with protein levels (Supplementary Figure 6). The latter constitutes one of the most important strengths of proteomic assessment of patient tissue. Nevertheless, combined assessment of both RNA and protein data allows a much more thorough insight into the tissue, as the coverage of transcriptomic analysis still is much broader – in this study, RNAseq identified 3.5-fold more targets than LC-MS/MS (Fig. 1D). In certain tissues, detection of proteins is more difficult than others, as demonstrated by the lower number of detected proteins in AT compared to the other tissues (Fig. 1D). Also, it is well-established that certain proteins are highly difficult to detect using LC-MS/MS, such as cytokines with low abundance [66]. In such cases, RNAseq data can significantly aid data interpretation. Hence, transcriptomics and proteomics represent complementary rather than redundant viewpoints that can be used to assess different questions (Fig. 3I).

While the recently available large-scale proteomic studies of human STS are very interesting and highly valuable [49,67], there are important limitations to the chosen approaches that our study addresses: firstly, these analyses are based on ‘tumor-enriched’ bulk approaches (i.e. containing up to 30 % of surrounding normal tissue per sample), secondly, neither of these studies has included specifically defined matched adjacent NT, which precludes identification of targets that specifically allow differentiation of tumor from its native surroundings. Thirdly, complementary RNAseq data is available only for a very small subset of 25 angiosarcoma, which limits assessing the combined power of transcriptome and proteome analysis. Finally, only 8 cases of fibroblastic STS were included in these large cohorts, which heavily limits the available data for these very rare tumors. Here, our study across 30 feline FSA provides highly valuable data to assess expression of candidate targets on protein and RNA level across three normal tissues that these tumors need to frequently be distinguished from, with relevance also for human STS (Fig. 2, Fig. 3). As such, this approach allows identification of tumor-specific diagnostic markers, which is of specific relevance also for the STS field, where correct diagnosis remains a challenge, especially so in the case of adult FSA, an exceedingly rare and aggressive tumor subtype lacking any specific marker of differentiation and represents a diagnosis of exclusion [4]. Moreover, our data is of specific value also with regards to development of targeted therapeutic approaches for STS, including strategies to selectively enrich radionuclides, fluorescent dyes, cytostatics, or CAR-T cells in the tumor using specific ligands [[68], [69], [70], [71]]. We anticipate refinement, validation and preclinical development of such targets in follow-up projects to support translating these insights into clinical practice.

In contrast to human STS, where molecular diagnosis allows classification of >100 different STS subtypes, diagnosis of veterinary STS entities is still largely based on histomorphology – especially so for feline tumors. This lack of granularity, combined with the inherent difficulty of diagnosing STS based on histomorphology alone [4], likely contributes to diagnostic inaccuracy and failure to identify existing subtypes that may also differ with regards to clinical prognosis. Interestingly, on the RNA level, we identified immune-mediated features and neuronal expression signatures as distinctive features between highly vs lowly aggressive behaving tumors, respectively (Fig. 4A-C). Moreover, expanding the analysis to the full dataset, we identify three subsets of tumors, C1 – C3. C1 is characterized by a fibroblastic gene expression pattern, consistent with the suggested fibroblastic origin of these tumors. Meanwhile, tumors in the C2 subset exhibit a neuronal expression signature, and the C3 subset features high expression of factors involved in immune regulation (Fig. 4E-H). With extensive expression of immunosuppressive targets, tumors of the C3 subset may be amenable to immune-checkpoint blockade. C2 are reminiscent of the group of peripheral nerve sheath tumors (PNSTs). In both humans and cats PNSTs come in different clinical flavors: they can range from benign subtypes, such as Schwannomas, to highly malignant tumors [4,72]. Importantly however, feline PNSTs that present histologically malignant features have never been documented to metastasize, suggesting these tumors in cats to behave in a more benign fashion than in humans [72]. In sharp contrast to this, ‘classic’ feline FSA are highly aggressive tumors that are characterized by poorly defined tumor margins, a high tendency to infiltrate surrounding tissue, form tumor extensions and satellite lesions and frequently display a high grade of malignancy, with a moderate metastatic rate [[73], [74], [75], [76], [77], [78], [79]], a range of local recurrence following surgical resection between 11-80 % and reported median survival times from 390 – 901 days [[79], [80], [81]], resulting in a very guarded prognosis. As such, the identification of a subset of feline FSAs with neuronal-like expression pattern that may coincide with more benign clinical behavior is relevant, if validated in further studies. ssGSEA of the proteomic data between highly and lowly aggressive tumors suggests a possible connection between the benign behavior of these neuronal-like tumors with a significant enrichment of inflammatory responses and activation of the complement and coagulation cascades (Fig. 4D). Interestingly, activation of the complement and coagulation system in human DDLPS has very recently been described to be associated with a significantly longer local recurrence-free survival [82]. Though these tumors in general display less tumor-infiltrating lymphocytes than tumors with low activation scores in these pathways, the complement system is an innate immune defense mechanism that precedes activation of adaptive immunity and deficiency therein impairs both B- and T-cell responses [83]. It is important to highlight here, that RNA and protein data yield opposing information regarding activation of several pathways, including the complement system. This is a feature that has only recently come to attention specifically also in STS, in the wake of more extensive proteomic analysis of patient tissues and may explain some of the discrepancies with previous literature linking high transcriptomic levels of complement activation with tumor malignancy [83]. As proteins represent the functional workunits of a cell, proteomic data might represent more faithfully the actual activation status of given pathways. Hence, these data suggest the existence of several molecular subtypes of feline FSA that also differ with regards to their clinical behavior, with immune-infiltrated tumors expressing high levels of checkpoint inhibitors and thus potentially amenable for treatment with ICI, and neuronal-like tumors associated with a potentially better clinical outcome. Moreover, this clearly highlights the need for more refined molecular diagnostic approaches to improve classification and diagnosis of feline FSA.

To assess the potential of feline FSA as clinically amenable models to assess therapeutic strategies in a structured manner to inform novel therapeutic approaches for human patients, we analyzed to what extent molecular features are conserved between feline and human data. Comparison of our data with the proteomic dataset on FSA and MFS by Tang *et al* [49] revealed striking similarities between the two species and demonstrated the use of our feline dataset to identify and prioritize potential tumor-targets conserved across both species (Fig. 5A and B). Moreover, by leveraging well-documented differences in clinical behavior between canine and feline FSA, we identified selective enrichment of DNA repair pathways in feline FSA and demonstrated a connection with tumor aggressiveness also in human STS (Fig. 5 D - H).

The established standard treatment for feline FSA is radical surgical excision. However, in a substantial number of feline FSA patients, satisfactory tumor control cannot be achieved with surgery alone. Additional therapeutic strategies using systemic treatment could be highly beneficial – particularly also in the metastatic setting - but is complicated by the high chemoresistance of feline FSA. To identify therapeutic vulnerabilities for neo-/adjuvant use to treat feline FSA, we selected a series of clinically available drugs based on the molecular features identified above for assessment of cell sensitivity of feline FSA cell lines. This validated the striking dependence of FSA on processes involving RNA transcription (Actinomycin, Doxorubicin) as well as mitotic spindle and G2M checkpoint activation (Vinca-Alkaloids) and uncovered a potential vulnerability of FSA towards ATR and PARP inhibition (Fig. 5I - M). Neo- and adjuvant treatment with the Doxorubicin-stereoisomer Epirubicin combined with surgery demonstrated superior tumor-free survival rates and disease-free interval compared to historic controls in 21 cats [84]. Though not widely used currently, Actinomycin has been used in cats in the context of a rescue protocol for feline lymphoma [85], and Vinca-Alkaloids are clinically used in cats for other malignancies, but none of these has undergone thorough clinical assessment in the context of FSA. Interestingly, DNA repair pathways have been identified as potentially druggable targets in a subset of human STS and have sparked currently running clinical trials [61,62]. To our knowledge, DNA repair inhibitors have not yet been assessed in the context of feline FSA or indeed any feline tumor. As such, our results uncover the therapeutic potential of ATR and PARP inhibition in the context of feline FSA for further clinical assessment.

Collectively, the results presented herein will serve as a starting point for further studies specifically on feline FSA and its potential as a clinically amenable model for adult FSA. Better understanding of the biology driving these tumors will promote the development of novel diagnostic and therapeutic approaches to benefit patients from both species.

## Data and code availability

The mass spectrometry proteomics data have been deposited to the ProteomeXchange Consortium via the PRIDE [86] partner repository with the dataset identifier PXD055198. The RNA sequencing raw data was submitted to the GEO repository and is available under the accession id GSE275872. All other data supporting our findings is contained in the manuscript and in the supplementary figures and tables.

## CRediT authorship contribution statement

**Mikiyo Weber:** Writing – review & editing, Writing – original draft, Visualization, Validation, Resources, Methodology, Investigation. **Daniel Fuchs:** Writing – review & editing, Visualization, Validation, Software, Resources, Methodology, Investigation, Formal analysis, Data curation. **Amiskwia Pöschel:** Writing – review & editing, Visualization, Methodology, Investigation. **Erin Beebe:** Writing – review & editing, Supervision, Methodology, Formal analysis, Data curation. **Zuzana Garajova:** Methodology, Investigation. **Armin Jarosch:** Conceptualization, Investigation, Resources, Writing – review & editing. **Laura Kunz:** Writing – review & editing, Resources, Methodology, Investigation. **Witold Wolski:** Writing – review & editing, Software, Methodology, Investigation. **Lennart Opitz:** Writing – review & editing, Software, Methodology, Data curation. **Franco Guscetti:** Writing – review & editing, Validation, Methodology, Investigation. **Mirja C. Nolff:** Writing – review & editing, Writing – original draft, Supervision, Investigation, Formal analysis, Conceptualization. **Enni Markkanen:** Writing – review & editing, Writing – original draft, Supervision, Project administration, Investigation, Funding acquisition, Formal analysis, Data curation, Conceptualization.

## Declaration of competing interest

The authors declare that they have no known competing financial interests or personal relationships that could have appeared to influence the work reported in this paper.

## Appendix Supplementary materials

Image, application 1

Image, application 2
