# Supplementary material for: Transcriptomic and proteomic profiling identifies feline fibrosarcoma as clinically amenable model for aggressive sarcoma subtypes
Source: Neoplasia. 2024 Dec 15;60:101104. doi: 10.1016/j.neo.2024.101104 (PMC11713505; doi:10.1016/j.neo.2024.101104)
Supplement: Supplementary file 2 [file mmc2.pdf]

Supplementary Figures

Transcriptomic and proteomic profiling identifies feline fibrosarcoma as clinically amenable model for aggressive sarcoma subtypes

Mikiyo Weber, Daniel Fuchs, Amiskwia Pöschel, Erin Beebe, Zuzana Garajova, Laura Kunz, Witold Wolski, Lennart Opitz, Franco Guscetti, Mirja C. Nolff and Enni Markkanen

Supplementary Figure 1

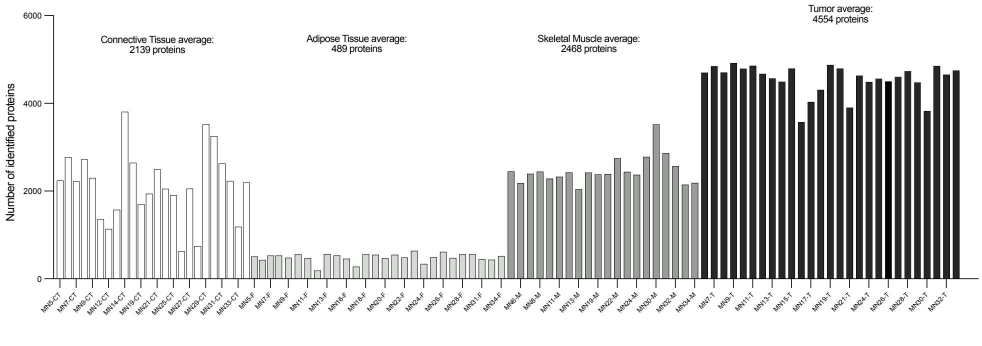

Supplementary Figure 1: Overview of number of proteins identified in each sample by LC-MS/MS.

Supplementary Figure 2

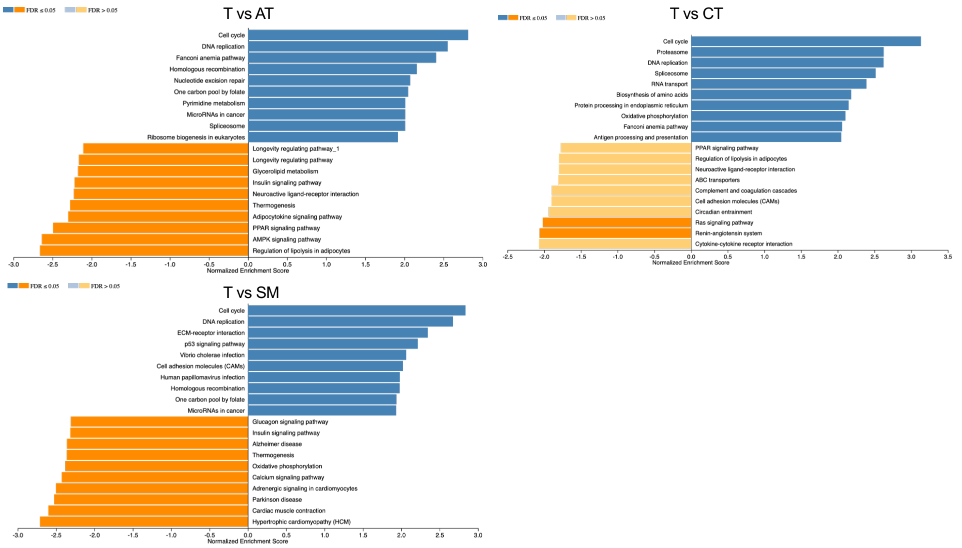

Supplementary Figure 2: GSEA of significantly differing transcripts between T and respective NT. GSEA was performed using the KEGG pathway with only the significant transcripts (FDR < 0.05).

Supplementary Figure 3

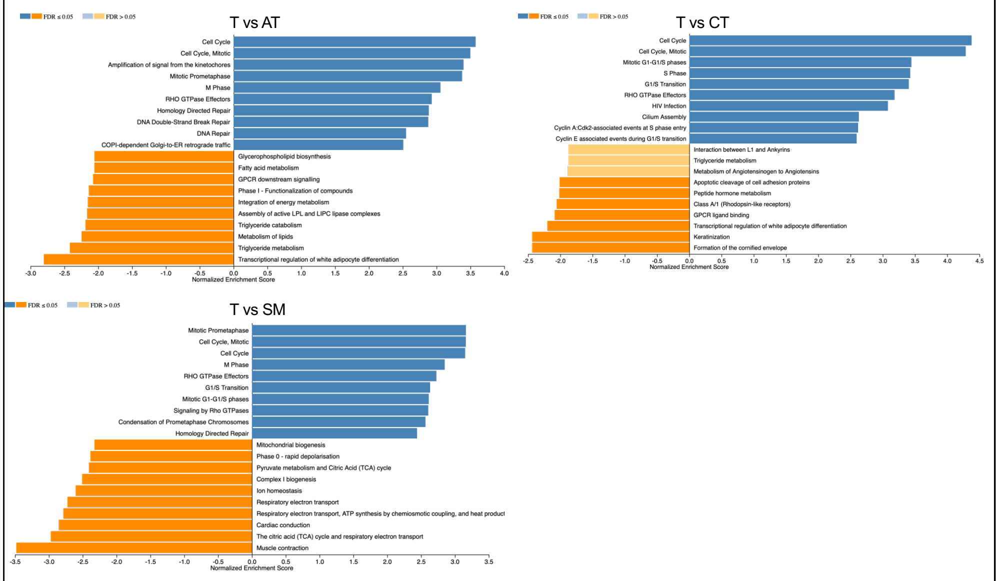

Supplementary Figure 3: GSEA of significantly differing transcripts between T and respective NT. GSEA was performed using the Reactome pathway with only the significant transcripts (FDR < 0.05).

Supplementary Figure 4

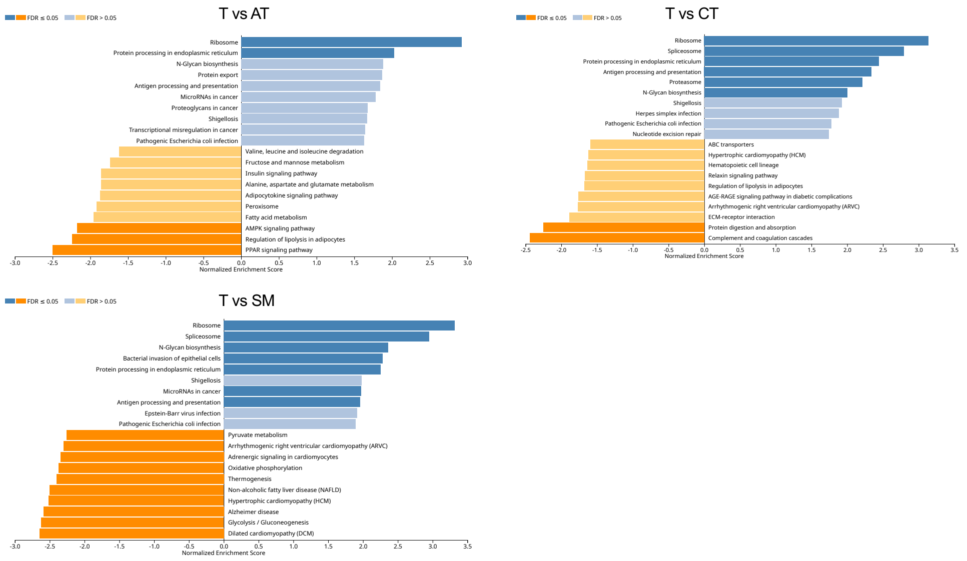

Supplementary Figure 4: GSEA of significantly differing proteins between T and respective NT. GSEA was performed using the KEGG pathway with only the significant proteins (FDR < 0.05).

Supplementary Figure 5

**Overlap Results**						
**Gene Set Name**	# Genes in Gene Set (K)	Description	# Genes in Overlap (k)	k/K	p-value	FDR q-value
**HALLMARK_MITOTIC_SPINDLE**	199	Genes important for mitotic spindle assembly.	18	0.0905	1.72E-11	4.68E-10
**HALLMARK_E2F_TARGETS**	200	Genes encoding cell cycle related targets of E2F transcription factors.	18	0.0900	1.87E-11	4.68E-10
**HALLMARK_G2M_CHECKPOINT**	200	Genes involved in the G2/M checkpoint, as in progression through the cell division cycle.	11	0.0550	1.97E-05	3.28E-04
**HALLMARK_MYC_TARGETS_V2**	58	A subgroup of genes regulated by MYC - version 2 (v2).	6	0.1034	4.98E-05	5.75E-04
**HALLMARK_EPITHELIAL_MESENCHYMAL_TRANSITION**	200	Genes defining epithelial-mesenchymal transition, as in wound healing, fibrosis and metastasis.	10	0.0500	1.03E-04	5.75E-04
**HALLMARK_GLYCOLYSIS**	200	Genes encoding proteins involved in glycolysis and gluconeogenesis.	10	0.0500	1.03E-04	5.75E-04
**HALLMARK_HYPOXIA**	200	Genes up-regulated in response to low oxygen levels (hypoxia).	10	0.0500	1.03E-04	5.75E-04
**HALLMARK_INFLAMMATORY_RESPONSE**	200	Genes defining inflammatory response.	10	0.0500	1.03E-04	5.75E-04
**HALLMARK_TNFA_SIGNALING_VIA_NFKB**	200	Genes regulated by NF-kB in response to TNF [GeneID=7124].	10	0.0500	1.03E-04	5.75E-04
**HALLMARK_UNFOLDED_PROTEIN_RESPONSE**	113	Genes up-regulated during unfolded protein response, a cellular stress response related to the endoplasmic reticulum.	7	0.0619	3.12E-04	1.49E-03

Supplementary Figure 5: ORA using HALLMARK pathway of the 625 tumor-exclusive proteins.

Supplementary Figure 6

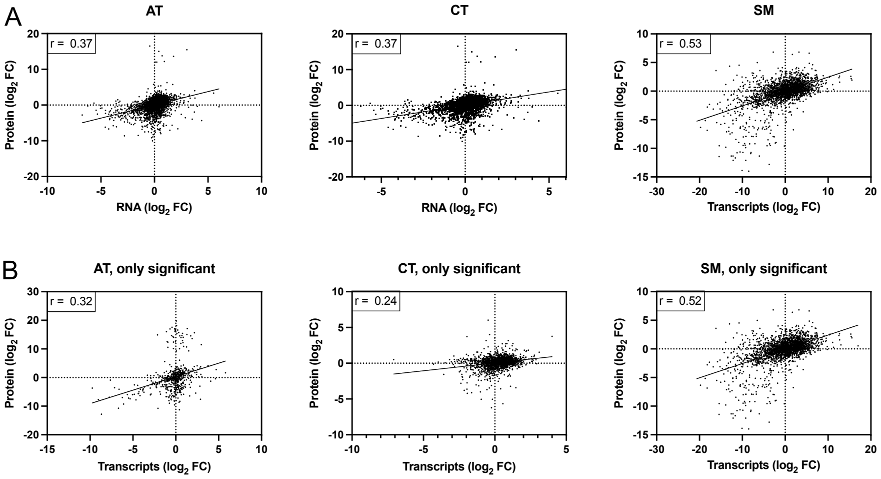

Supplementary Figure 6: Correlation analysis of proteomics versus transcriptomics. A) Correlation plot using the log_2(FC) of T vs AT, T vs CT and T vs SM in proteomics and transcriptomics. B) as A) but using only significantly changing targets (FDR<0.05).
